# Complete Genome Sequence of α-1,3-Glucanase-Producing Strain *Paracoccus mutanolyticus* RSP-02

**DOI:** 10.1128/MRA.01095-18

**Published:** 2018-10-11

**Authors:** Ravi Naga Amrutha, Abhishek Shrivastav, Reddy Shetty Shravani, Sudheer Kumar Buddana, Uday Deshpande, Reddy Shetty Prakasham

**Affiliations:** a Medicinal Chemistry and Biotechnology, CSIR-Indian Institute of Chemical Technology, Hyderabad, India; b Bioserve, A Reprocell Company, Hyderabad, India; c Institute of Bioinformatics and Applied Biotechnology (IBAB), Bengaluru, India; Queens College

## Abstract

A mutanase (α-1,3 glucanase)-producing bacterial strain of Paracoccus mutanolyticus was isolated from soil samples rich in cellulosic waste. Here, we report the whole-genome sequencing and annotation of P. mutanolyticus, which has a genome size of around 3.5 Mb and the potential to degrade water-insoluble α-1,3 glucans with an overall G+C content of 67.4%.

## ANNOUNCEMENT

Mutanases (α-1,3 glucanases) are the enzymes that hydrolyze mutan (water-insoluble α-1,3 glucan) secreted by tooth-colonizing oral streptococci, which is a key component in the development of dental caries (1–3). In an attempt to identify microorganisms that produce mutanase, a bacterial strain with the ability to degrade mutan was isolated from a soil sample rich in cellulosic waste collected at Sirpur Paper Mills in Kaghaznagar, Telangana, India. Biochemical and 16S rRNA sequence analysis indicated that the isolated strain is an aerobic, Gram-negative, nonmotile, spherical bacterium that belongs to the *Paracoccus* genus. The strain was further characterized for its ability to degrade mutan and was designated Paracoccus mutanolyticus RSP-02 (4).

A Paracoccus mutanolyticus culture was grown overnight at 37°C in nutrient broth, and DNA was extracted using a DNeasy UltraClean microbial kit (Qiagen). For whole-genome sequencing, purified DNA was fragmented to a size of 200 bp using Covaris M220 (Thermo Fisher Scientific). Next-generation sequencing (NGS) library preparation was performed using a NEBNext Ultra II DNA kit (New England Biolabs) and sequenced on a NextSeq 500 platform (Illumina, San Diego, CA). A total of 25,708,952 FASTQ reads were generated with an average read length of 150 bp. Reads were trimmed using Cutadapt ver1.17 software (5), and FASTQ files were analyzed using FastQC software (6) and generated a comprehensive quality control (QC) report. High-quality clean reads with a Phred score of >30 were selected for assembly using the CLC Genomics Workbench ver9.0 suite (Qiagen). A total of 694 contigs were generated after *de novo* assembly, with an average contig length (*N*_50_) of 77,019 bp, and the maximum contig length obtained was 202,391 bp. The generated contigs were mapped to the reference genome of Paracoccus yeei. The complete genome sequence of P. mutanolyticus comprises 3,592,357 bp with an overall G+C content of 67.4%. For annotation, a 3,592,357-bp genome sequence of Paracoccus mutanolyticus was submitted to the National Center for Biotechnology Information (NCBI) and analyzed with the Prokaryotic Genome Annotation Pipeline (PGAP) (7).

The genome of Paracoccus mutanolyticus contains a total of 3,889 coding sequences, 3,827 of which are functional genes and 62 genes encode RNA (50 tRNA, 9 rRNA, and 3 noncoding [ncRNA] genes). The genome of this isolate exhibited 1,492 pseudogenes. The occurrence of pseudogenes in the bacterial genome is not unusual, as it has been reported that in prokaryotes, the percentage of pseudogenes ranges from 1 to 8% of the total genome (8, 9). There are several possible reasons for the presence of a high number of pseudogenes in the bacterial genome, and the major reason for this could be mutational events occurring in bacterial genomes during evolutionary processes and genome assembly artifacts (9).

### Data availability.

This whole-genome shotgun project has been deposited in DDBJ/ENA/GenBank under the accession number CP030239. The version described in this paper is the latest version, CP030239.1. The raw FASTQ reads have been deposited in the NCBI SRA database under the accession number SRX4598341.
